# Use of simulation-based medical training in Swiss pediatric hospitals: a national survey

**DOI:** 10.1186/s12909-017-0940-1

**Published:** 2017-06-17

**Authors:** Martin Stocker, Kathryn Laine, Francis Ulmer

**Affiliations:** 1 Pediatric and Neonatal Intensive Care Unit, Children’s Hospital Lucerne, Spitalstrasse 16, CH-6000 Luzern, Switzerland; 2 Department of Pediatrics, University Hospital Lausanne, Lausanne, Switzerland; 3 Pediatric Intensive Care Unit, Children’s Hospital, University Hospital Berne, Bern, Switzerland

**Keywords:** Simulation-based medical training, Team training, Technical skills, Crisis resource management, Pediatrics, Inter-professional education

## Abstract

**Background:**

Simulation-based medical training (SBMT) is a powerful tool for continuing medical education. In contrast to the Anglo-Saxon medical education community, up until recently, SBMT was scarce in continental Europe’s pediatric health care education: In 2009, only 3 Swiss pediatric health care institutions used SBMT. The Swiss catalogue of objectives in Pediatrics does not acknowledge SBMT. The aim of this survey is to describe and analyze the current state of SBMT in Swiss pediatric hospitals and health care departments.

**Methods:**

A survey was carried out with medical education representatives of every institution. SBMT was defined as any kind of training with a mannequin excluding national and/or international standardized courses. The survey reference day was May 31st 2015.

**Results:**

Thirty Swiss pediatric hospitals and health care departments answered our survey (response rate 96.8%) with 66.6% (20 out of 30) offering SBMT. Four of the 20 hospitals offering SMBT had two independently operating training simulation units, resulting in 24 educational units as the basis for our SBMT analysis. More than 90% of the educational units offering SBMT (22 out of 24 units) were conducting in-situ training and 62.5% (15 out of 24) were using high-technology mannequins. Technical skills, communication and leadership ranked among the top training priorities. All institutions catered to inter-professional participants. The vast majority conducted training that was neither embedded within a larger educational curriculum (19 out of 24: 79.2%) nor evaluated (16 out of 24: 66.6%) by its participants. Only 5 institutions (20.8%) extended their training to at least two thirds of their hospital staff.

**Conclusions:**

Two thirds of the Swiss pediatric hospitals and health care departments are offering SBMT. Swiss pediatric SBMT is inter-professional, mainly in-situ based, covering technical as well as non-technical skills, and often employing high-technology mannequins. The absence of a systematic approach and reaching only a small number of healthcare employees were identified as shortcomings that need to be addressed.

**Electronic supplementary material:**

The online version of this article (doi:10.1186/s12909-017-0940-1) contains supplementary material, which is available to authorized users.

## Background

Simulation-based medical training (SBMT) is a powerful tool for delivering continuing medical education. There is mounting evidence supporting the effectiveness of this training methodology [1–5]. Recent publications report improved confidence and self-efficacy among training participants, as well as improved patient outcomes in Children’s Hospitals after implementation of SBMT programs [6–12]. Up until recently SBMT was scarce in continental Europe’s pediatric health care education [13]. This is contrasted by the medical education communities of North America, Australia and the United Kingdom which have embraced SBMT for some time [5, 14–16]. In 2009, a survey in German-speaking countries reported only 3 Swiss pediatric hospitals using SBMT [17]. Ten years ago, the Swiss medical graduate education community started using SBMT in both technical and non-technical domains of clinical medicine for Swiss medical students [18, 19]. The post graduate training program of the Swiss Pediatric catalogue of objectives released by the Swiss Institute of Medical Education (SIWF) supports nationally and/or internationally standardized courses in neonatal and pediatric resuscitation but does not include SBMT [20]. To date, there are no SIWF regulations or recommendations condoning or mentioning SBMT as an educational modality. As a consequence there is no established national accreditation program enforcing SBMT standards to which Swiss pediatric hospitals must adhere.

The framework of “simulation fidelity” can be used to describe SBMT [5, 21, 22]. The term “simulation fidelity” comprises technical (equipment), environmental and psychological aspects of SBMT. Equipment fidelity describes the degree to which simulation replicates reality. Often this aspect focuses on mannequin fidelity (low- versus high-technology mannequins) [21]. Environmental fidelity describes the extent of environmental reality in which the SBMT session takes place. The environmental fidelity of a designated simulation center needs to be distinguished from that of in-situ SBMT, which takes place in the actual clinical environment, where patients are normally treated. The degree of perceived reality on behalf of the participants is described by the term psychological fidelity [5]. Psychological fidelity is governed by a multitude of factors including equipment and environmental fidelity, content of the training session (e.g. perceived reality of the scenario) as well as team composition (randomly assembled teams versus ones that work together in real life) [5, 23]. Design and implementation strategies for SBMT are still evolving and best practices are not yet well established, but a systematic approach seems to be beneficial [2, 4, 8, 14, 23, 24]. The framework of Kern for curriculum development including needs assessment, educational and implementation strategies, evaluation and feedback describes a systematic approach for delivering continuing medical education [25].

The aim of this national survey is to describe and analyze the current state of SBMT in Swiss pediatric hospitals. We seek to analyze and compare established and successfully operating SBMT programs and elucidate current gaps in simulation-based pediatric medical education.

## Methods

For the purpose of this survey, we defined SBMT as any kind of health care provider related training using a mannequin in a contextualized clinically realistic scenario at the surveyed pediatric hospital. Nationally and/or internationally standardized courses such as Pediatric Advanced Life Support (PALS), European Pediatric Life Support (EPLS), Advanced Trauma Life Support (ATLS) Basic Life Support (BLS), or the Swiss neonatal resuscitation program (start4neo) were excluded from our SBMT definition, because these formats constitute standard training programs recommended for Swiss pediatric board certification and do not mandate the use of simulation technology.

### National survey

**Fig. 1:**
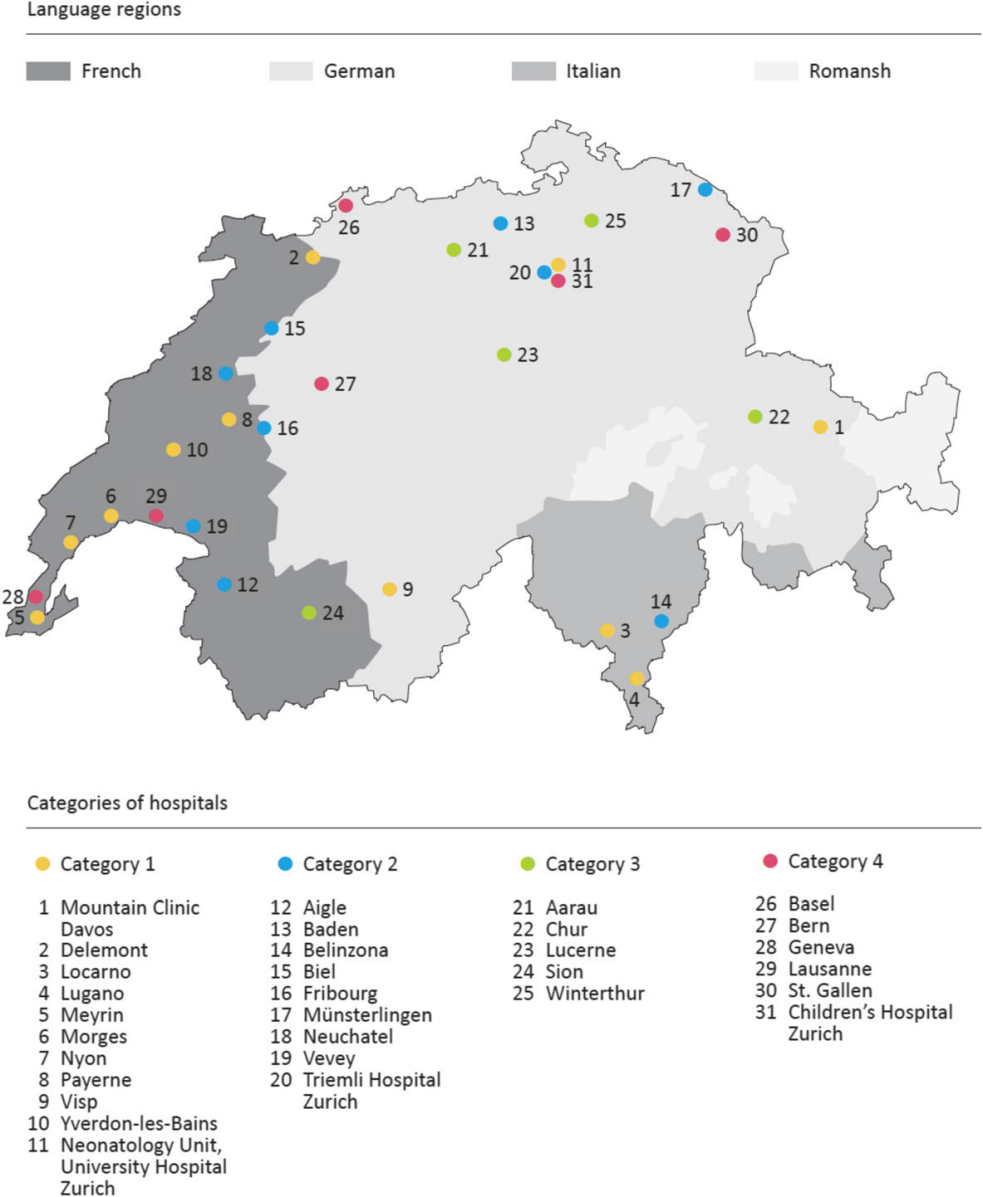
Geographic distribution of Swiss pediatric educational institutions

The Swiss Medical Association (FMH) recognizes 31 pediatric hospitals nationwide [26]. The SIWF categorizes these pediatric hospitals according to the degree of health care services they provide: Hospitals offering basic are defined as category 1; extended primary health care hospitals offering optional subspecialties are defined as category 2; category 3 offers a higher degree and category 4 offers the highest degree of educational specialization (referral academic hospitals) [20]. See Fig. 1 depicting the geographic distribution of Swiss pediatric hospitals. Staff in Swiss pediatric hospitals is commonly comprised of nurses with a wide range of experience (ranging from nursing students and nurses assistants to senior registered nurses and advanced practitioners), physicians (students, residents, fellows, and consultants), as well as allied health professionals including midwives, medics and occupational health practitioners.

Between June and August 2015 a national survey developed by the authors was carried out with representatives of all Swiss pediatric hospitals (categories 1 to 4). The survey reference date was May 31st 2015. Hospital representatives were approached by email or phone with a request to participate. Those who did not respond received a follow-up reminder within 2 to 4 weeks. We attempted to directly contact hospital staff in charge of simulation-based training. In the event that this was not practicable, we contacted the head of the pediatric department and requested to be referred to the respective educational staff person. Up to three representatives (physicians and/or nurses) were interviewed per training program. We asked for clarification when there were relevant discrepancies in the responses from representatives from within the same training program. The response rate was calculated by comparing the number of potential hospitals to the number of hospitals that responded to our survey. Consent was implied upon completion of the study questionnaire. According to the Swiss law regarding research including human beings educational surveys do not need to be approved by a Swiss Ethical Board [27]. This is in accordance to the published guidelines of the British Educational Research Association (BERA) [28].

### Questionnaire

The questionnaire was developed specifically for this survey and trialed on non-pediatric simulation instructors known to the authors. There were two main sections of the questionnaire: The first section was made up of five general questions that investigated present utilization of simulation-based training or future plans to do so, and in the second section followed by twenty questions that focused on how hospitals offer simulation-based training. The 20 questions were composed of three sub-sections and looked at how simulation-based training was carried out: 9 questions focusing on design and organization, 7 questions on SBMT participation and 4 questions on SBMT instruction. The main focus of the design and organization subsection was to evaluate the presence of a systematic approach according to the framework of Kern for curriculum development (training embedded within an educational curriculum, areas of training content, frequency of simulation-based training sessions, tools of team training, structured participant evaluation of the training sessions and research activities) [25]. The remaining 2 questions in this subsection examined simulation fidelity: equipment fidelity was queried regarding the use of low- and/or high-technology mannequins. Environmental fidelity was queried concerning in-situ simulation or training in a designated simulation center, or both. Psychological fidelity was not part of this survey because we did not interview training participants from the different simulation-based training programs. The 7 questions examining SBMT participants focused on inter-professional and/or multidisciplinary composition of participating individuals, percentage of hospital staff involvement in the 17 months leading up to our survey and SBMT time constraints (training hours counting as working hours and protected time for participants during SBMT sessions). The last 4 questions focused on SBMT time constraints for instructors (training hours as working hours, required overtime exceeding working hours, protected time during training session).

### Statistics

Completed surveys were entered into an excel spread sheet and tabulated. Descriptive analyses were used to compare answers. Institutions with high (>66%) versus low (<33%) training involvement of physician and nursing staff were compared and analyzed. Answers were compared using the two-tailed Fisher exact probability test. A *p*-value <0.05 was considered statistically significant with a confidence interval at 95%.

## Results

Of the 31 Swiss pediatric training hospitals recognized by the Swiss Medical Association (FMH) 30 answered our survey (response rate 96.8%). Twenty of the 30 surveyed hospitals (66.6%) were offering SBMT within their departments. None of the hospitals were outsourcing SBMT to outside hospitals. Two hospitals were planning to introduce simulation training within the next two years. Both geographic location relative to spoken language (German versus French versus Italian parts of Switzerland, see Fig. 1) and category of pediatric hospital (categories 1 to 4) were independent of SBMT prevalence: Five out of ten category 1 hospitals offered SBMT (one category 1 hospital failed to respond to our survey); six out of nine category 2 hospitals -, four out of five category 3 hospitals - and five out of six category 4 hospitals offered SBMT. Several of the category 4 hospitals were running departmentalized educational programs that were operating completely independent of one another, e.g. general pediatric ward, neonatal and pediatric intensive care units, and pediatric emergency departments. Four of the 20 hospitals offering SMBT had two independently operating training simulation units. As a result we decided to use these 24 units as the basis for our SBMT analysis, rather than the 20 hospitals they originated from. Fourteen of the 24 units (58.3%) currently offering SBMT were planning to enlarge their programs within the next two years, but only 9 of these 14 units anticipated having sufficient support to pursue such a plan.

### Design and organization of simulation training

**Fig. 2:**
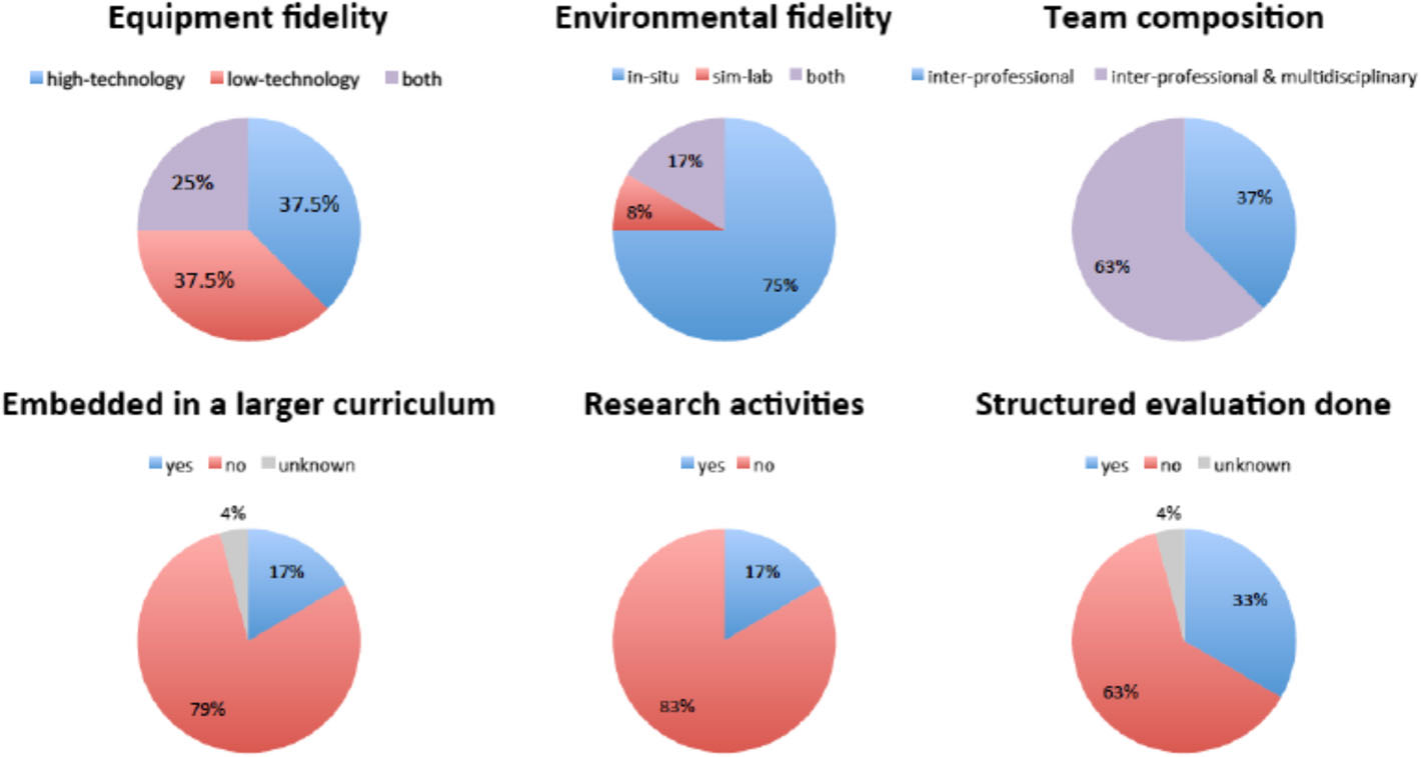
Design and organization of Simulation-based medical training

Fifteen out of 24 educational units (62.5%) were offering low-technology simulation training. An equal number (15 out of 24) of units offered simulation training with high-technology equipment. One quarter (6 out of 24) of the units reported using both low- and high-technology equipment. More than 90% (22 out of 24 units) were offering in-situ training and 25% (6 out of 24 units) in a designated simulation center on site. The frequency of training offered (at least once a month) in 2014 increased from 54.2% (13 out of 24) to 75.0% (18 out of 24 units) in 2015, without the difference approaching statistical significance (*p* = 0.22). The 24 units focused their training on the following skills: Technical skills (23 out of 24 units: 95.8%), communication (23 out of 24: 95.8%), leadership (21 out of 24: 87.5%) and role clarity (19 out of 24: 79.2%) ranked among the top skills that were prioritized. In 18 of the 24 educational units SBMT was the only format for team training. In a majority of units (19 out of 24: 79.2%) the training was not embedded within a larger educational curriculum. Only one third (8 out of 24) of the units offering SBMT enabled simulation participants to formally evaluate their training in a standardized and structured fashion. Research activities rarely (4 out of 24 units: 16.6%) accompanied SBMT. The principal results are visualized in Fig. 2. Raw data are available as an on-line supplement (see Additional file 1).

### Participants

**Fig. 3:**
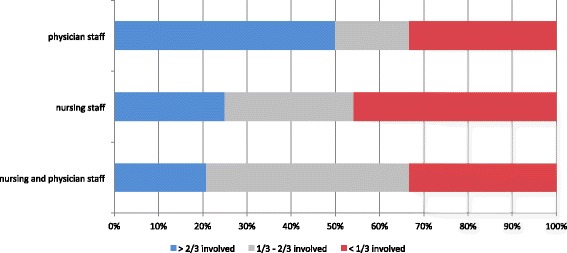
Simulation-based medical training involvement of physician and nursing staff (nurses and mid-wives) between January 2014 and June 2015 (17 months); 24 educational units = 100%

All units (24 out of 24) offering SBMT included inter-professional teams (at least two professions, including mainly physicians, nurses, mid-wives) (Fig. 2). Fifteen of the 24 units (62.5%) involved multi-disciplinary physicians (at least two different disciplines) consisting of general pediatricians (12 out of 24 units: 50%), pediatric emergency physicians (12 out of 24: 50%), neonatologists (8 out of 24: 33%), anesthetists (8 out of 24: 33%), pediatric intensivists (7 out of 24: 29.2%) and pediatric surgeons (7 out of 24: 29.2%). Half of the units (12 out of 24) had involved at least two thirds of their physicians, whereas only 25% (6 out of 24) of the units reached more than two thirds of their nursing staff in the training in the preceding 17 months (since the beginning 2014) (Fig. 3). In two thirds (16 out of 24) of the units training was mandatory for employed healthcare providers who had been selected to participate. For a majority of participants (20 out of 24: 83.3%) time spent practicing SBMT counted toward work time and at half (12 out of 24) of the units participants enjoyed protected time during their SBMT sessions. Raw data are available as additional on-line supplement (see Additional file 1).

### Instructors

Seventy-one instructors were engaged in SBMT at 24 units. Only a minority of them (22 out of 71: 31%) had simulation education mentioned as part of their job description and only 7 of the 71 instructors had a designated workload of 10% or more allocated for simulation training according to their contract. Seventy percent (50 out of 71) of the instructors worked excess hours outside of what was expected of them to keep their SBMT programs functioning and operational: 54.9% (39 out of 71) worked up to five hours, 5.6% (4 out of 71) up to 10 h, 7% (5 out of 71) up to 20 h and 2.8% (2 out of 71) more than 20 h per month in excess of what was respected of them. 43.7% (31 out of 71) of instructors enjoyed protected time designated exclusively toward SBMT. Raw data are available as additional on-line material (see Additional file 1).

### Comparison of units with high (>66%) versus low (<33%) training involvement of physician and nursing staff (Table 1)

Five units enabled more than two thirds of their physician and nursing staff to participate in SBMT within the preceding 17 months (blue bar in Fig. 3). Eight units offering SBMT reached less than one third of their physician and nursing staff (red bar in Fig. 3). All units with high (defined as >66%) involvement of their staff were located in the French part of Switzerland, whereas 7 out of 8 units with low (defined as <33%) involvement were located in the German part of Switzerland. The use of high-technology equipment was paralleled by low staff involvement. Similarly, structured participant SBMT evaluation was sought significantly more often in institutions with low staff involvement. Instructors in institutions with low staff involvement enjoyed significantly more protected time to prepare and promote their SBMT with instructor overtime significantly exceeding 5 h.

**Table 1:** Comparison of institutions with high (>66%) versus low (<33%) percentages of physician and nurse training involvement

Areas with significant differences	High participation	Medium participation	Low participation	*p*-value low vs high
Geographical area: French part of Switzerland	5/5 (100%)	7/11 (64%)	1/8 (13%)	0.001
Equipment: High-technology	2/5 (40%)	5/11 (45%)	8/8 (100%)	0.034
Structured evaluation by participants	0/5 (0%)	3/11 (27%)	6/8 (75%)	0.02
Instructors: protected time	3/20 (15%)	10/28 (36%)	19/26 (73%)	<0.001
Instructor’s overtime exceeding 5 h	0/20 (0%)	2/27 (7%)	9/22 (41%)	0.001

Legend Table 1: Only results with statistical significance of differences between high versus low participation are shown. Participation within the 24 educational units was not dependent on the category of the pediatric hospital (categories 1 to 4)

## Discussion

Two thirds of Swiss pediatric hospitals offered SBMT in 2015. Our survey reveals a significant surge in the use of SBMT from 3 institutions in 2009 to 20 out of 31 institutions in 2015. This increase is in accordance with the recent medical education literature showing that simulation can be a powerful tool for continuing medical education and patient safety [1–5, 14]. Our goal is to stimulate discussion and co-operation between the institutional and national stakeholders of pediatric training and to improve continuing medical education promoting a better understanding of this powerful training methodology. We found the use of SBMT not to be dependent on educational category of the hospital. Swiss pediatric health care units with established SBMT predominantly used in-situ based training focusing on technical as well as non-technical skills involving of inter-professional and multidisciplinary participants. Only a minority of units applied a systematic approach and offered structured SBMT programs consisting of larger educational curriculums, structured participant evaluations and research activities. Furthermore, only a few units (*n* = 5) were able to extend SBMT to more than two thirds of their inter-professional staff. This knowledge shall serve as a guide for designing, improving and implementing future SBMT programs throughout and beyond Switzerland. The lack of a systematic approach and the need to involve a higher percentage of hospital staff constitute areas warranting improvement. More awareness, discussion and co-operation between institutional and national stakeholders in pediatrics are needed to promote SBMT as a unique and powerful experiential training methodology.

The fact that the majority of units offer SBMT in the in-situ setting leads us to assume that in-situ SBMT is best suited to meet the educational needs of Swiss pediatric training hospitals. Many reasons point to the advantages of in-situ simulation over laboratory simulation in a designated simulation center: Simulation experiences within the actual clinical environment, using real equipment and allowing for realistic team compositions enable the incorporation of technical, social and cultural contexts into the training format and may benefit psychological fidelity [23]. Lack of space in the clinical work environment is one of the main problems accompanying the implementation of in-situ simulation. Expenses on the other hand are usually lower compared to training programs that operate a designated simulation center. However focus and technical options of a designated simulation center may differ significantly when compared to in-situ SBMT [29]. The increased environmental fidelity provided by in-situ simulation bares an additional advantage to detect latent safety threats favorably impacting patient safety in a preventative fashion [7, 11, 24, 30]. Adverse, critical events secondary to latent safety threats occur at low frequency, but carry the potential for high acuity in Children’s hospitals [24, 31, 32]. Hence, in-situ simulation may play a key role in Children’s hospitals for improving patient safety as well as promoting inter-professional experiential learning [6, 7, 9, 11, 24, 30].

Teamwork is essential for optimal care and patient safety, especially in high-risk working areas where fluctuating groups of health care professionals congregate, as is the case in neonatal and pediatric critical care units [33–35]. Effective teamwork does not occur naturally, it must be acquired [36–38]. Communication failures e.g. are alarmingly common in health care and pose an obstacle for effective teamwork [37, 39]. However, nurses and physicians often hold different attitudes and opinions on how effective teamwork should be implemented [40, 41]. Literature reviews show that simulated team training can promote teamwork by improving non-technical skills such as leadership behaviors and communication [42–46]. Our results show that this aspect is not only recognized but in fact emphasized within the SBMT programs: technical skills as well as non-technical skills (communication, leadership, role clarity) were top priorities in the vast majority of units offering inter-professional and multidisciplinary training sessions. Inter-professional education is viewed as an emerging concept on how to improve inter-professional teamwork [47–50]. SBMT offers the opportunity to acquire inter-professional teamwork skills when learning on the job is too risky [10, 24, 50]. To date, none of the Swiss guidelines for continuing education recommend SBMT as an educational modality.

Only one third of the units were evaluating their training programs through the eyes of the simulation participants in a standardized and structured format. In addition, a mere 4 of 24 SBMT programs were part of a larger educational curriculum. Research activities were rare (16.6%). This stands in contrast to the fact that the evaluation of any training program is an essential component to ensure feedback and maintain quality [25]. Research activities are a good vehicle for disseminating information and transparency [4, 24, 25]. Some educational research has suggested that actions aiming to improve patient safety in Swiss pediatric hospitals, such as SBMT, could serve as a measure to investigate and elucidate the state of patient safety in Swiss pediatric institutions [24, 31]. Accordingly, there is evidence that clinical relevant SBMT favorably impacts error management and is an effective way to improve patient safety [51–54]. Our survey was limited to the implementation of SBMT within a larger educational curriculum. The link between SBMT’s impact on broader patient safety issues, such as attitudes toward medical errors and adverse events, patient safety committees and morbidity rounds was not investigated. The Components of a systematic approach should, among other things, include a needs assessment, the determination of goals and objectives and an evaluation involving feedback. These are mandatory steps for any high-quality curriculum development [25]. In the absence of a systematic approach, the risk for erratic training content increases. An isolated SBMT program that is not embedded within a larger educational curriculum runs the risk of having low impact and not fulfilling its intended goals.

In this study, five units reported having included more than two thirds of their hospital staff (physicians and nurses) in their SBMT within the preceding 17 months, whereas eight units reported having involved less than one third. Comparing these two groups of hospitals, we identified significant differences: First, the use of high-technology simulation was associated with a low penetration of hospital staff. This is in accordance with the literature that high-technology simulation is much more resource intense compared to low-technology [21]. Despite the privilege of enjoying significantly more protected time and accepting excess working hours, instructors using high-technology simulation managed to train only a small proportion of the hospital staff. This confirms the value of needs assessments and the importance of having clearly defined goals when making the decision whether to apply low or high-technology simulation training [25, 55]. The paucity of structured evaluation among hospitals with a high rate of SBMT penetration for hospital staff compared to the group with low involvement suggests that a lack of resources inhibits the implementation of a systematic approach. All units with high training involvement of staff were located in the French part of Switzerland, whereas units with low training involvement of staff were predominantly situated in the German part of Switzerland (compare Fig. 1 and Table 1). Given the cultural differences between the two parts of Switzerland, we hypothesize that the cultural context plays a considerable role in the observed distribution. Hospitals situated in the French part of Switzerland with high training involvement of staff but limited assessment can be interpreted as using SBMT as a part of their regular educational practice accepting structural and methodical limitations, whereas hospitals situated in the German part of Switzerland with low training involvement of staff, more sophisticated technology and better staff assessment are using SBMT in a more structured setting, focusing on educational methods and learning outcome assessment. The literature regarding culture differences and medical education is sparse, but there are hints that cultural background may play an important role regarding acceptance of SBMT and safety culture in a hospital [56–60]. There were no other significant differences between the two groups. Remarkably, the level of involvement of hospital staff was not dependent on size or category of the hospital. Therefore, the assumption that SBMT offered at university hospitals is more sophisticated and extended to a larger portion of hospital staff, compared to smaller pediatric hospitals, is not valid for SBMT in Switzerland. To date there is very little data on the prevalence of medical simulation training at both the national level as well as at the level of the respective specialty or subspecialty. In their international survey on health care education Qayumi et al. reported in 2014 that two-thirds of responding institutions deemed simulation as integral to their curricula [61]. Okuda et al. found that 91% of emergency medicine residency programs in the United States used some form of simulation in 2008 [62]. By comparison the 67% prevalence of SBMT among Swiss pediatric hospitals with involvement of only a minority of hospital staff seems relatively poor.

There are some limitations in our survey: Psychological fidelity and effectiveness of the simulation-based training were not assessed as a part of this survey, as they exceed the scope of our questionnaire. Therefore we are unable to draw any conclusions regarding quality of the surveyed training. Similarly, we did not investigate assessment methodology, assessment frequency and assessed quality of individual and/or team performance during SBMT. Secondly, analysis and comparison of units with high versus low training involvement of physician and nursing staff involved a relatively small number of units. Nevertheless, our results objectify the importance of systematically designing and implementing SBMT. Thirdly, our definition of simulation limited our assessment to simulation training involving mannequins and did not consider other formats of simulation training such as role-play or standardized patients. Finally, as an inherent limitation of every survey research method, answers to a survey may not reflect the complete truth due to personal prejudice and the subjective assessment by the individuals who completed it.

## Conclusions

We observed a marked increase of SBMT in recent years in Swiss pediatric hospitals. The training was predominantly in-situ based, inter-professional and multidisciplinary. Focus of the training was a combination of technical and non-technical skills with communication, leadership and role clarity serving as top priorities. Only a minority of hospitals applied systematic approaches to their SBMT programs consisting of educational curriculums, structured participant evaluations and research activities. Only a minority of hospitals were able to include more than two thirds of their inter-professional staff. The lack of a systematic approach and the need to involve a higher percentage of hospital staff constitute areas warranting improvement. More awareness, discussion and co-operation between institutional and national stakeholders in pediatrics are needed to promote SBMT as a unique and powerful experiential training methodology worthy of incorporation into the Swiss catalogue of objectives in pediatrics.

## Additional file

Additional file 1: Raw data of the 24 hospitals/units using SBMT in 2015. (DOCX 33 kb)

## Abbreviations

ATLS: Advanced Trauma Life Support
BLS: Basic Life Support
EPLS: European Pediatric Life Support
FMH: Swiss Medical Association
PALS: Pediatric Advanced Life Support
SBMT: Simulation-based medical training
SIWF: Swiss Institute of Medical Education
