# Supplementary material for: Use of simulation-based medical training in Swiss pediatric hospitals: a national survey
Source: BMC Med Educ. 2017 Jun 17;17:104. doi: 10.1186/s12909-017-0940-1 (PMC5473998; doi:10.1186/s12909-017-0940-1)
Supplement: Additional file 1: — Raw data of the 24 hospitals/units using SBMT in 2015. (DOCX 33 kb) [file 12909_2017_940_MOESM1_ESM.docx]

Additional file to the manuscript "Use of simulation-based medical training in Swiss pediatric hospitals: A national survey"

**Additional file 1**

Raw data of the 24 hospitals/units using SBMT in 2015

How do Swiss pediatric health care educational units conduct Simulation-based medical training (SBMT)? Raw data of the 24 hospitals/units using SBMT in 2015:

| **Design and organization** | | |
| --- | --- | --- |
| Equipment used*   - low-fidelity - high-fidelity | 15/24  15/24 | 62.5%  62.5% |
| Setting*   - in-situ - designated simulation-center | 22/24  6/24 | 91.6%  25.0% |
| How often was SBMT offered from January to June 2015 (5 months)   - ≥ every two weeks - ≥ every month - ≥ every 2 months - < every 2 months | 5/24  18/24  22/24  2/24 | 20.8%  75.0%  91.6%  8.3% |
| How often was SBMT offered in 2014   - ≥ every two weeks - ≥ every month - ≥ every 2 months - < every 2 months | 4/24  13/24  20/24  4/24 | 16.6%  54.2%  83.3%  16.6% |
| Which domains does your SBMT focus on   - technical skills - communication - situational awareness - leadership - role clarity - error management - others | 23/24  23/24  13/24  21/24  19/24  10/24  6/24 | 95.8%  95.8%  54.2%  87.5%  79.2%  41.7%  25.0% |
| Do you offer team training other than SBMT?   - yes - no | 6/24  18/24 | 25.0%  75.0% |
| Is the SBMT embedded within a larger curriculum   - yes - no - unknown | 4/24  19/24  1/24 | 16.6%  79.2%  4.2% |
| Is SBMT subject to a structured participant evaluation   - yes - no - unknown | 8/24  15/24  1/24 | 33.3%  62.5%  4.2% |
| Are research activities ongoing in conjunction with SBMT   - yes - no | 4/24  20/24 | 16.6%  83.4% |
| **Participants** | | |
| Participants are^€^   - inter-professional - inter-professional and multidisciplinary | 24/24  15/24 | 100%  62.5% |
| Participating physicians come from   - general pediatrics - pediatric intensive care unit (PICU) - neonatal intensive care unit (NICU) - pediatric emergency medicine - pediatric anesthesia - pediatric surgery - others | 12/24  7/24  8/24  12/24  8/24  7/24  5/24 | 50.0%  29.2%  33.3%  50.0%  33.3%  29.2%  20.8% |
| Percentage of pediatric physicians who participated from January 2014 to June 2015 (17 months)   - < 10% - 10-33% - 34-66% - 67-100% | 3/24  5/24  4/24  12/24 | 12.5%  20.8%  16.6%  50.0% |
| Percentage of nurses^£^ who participated from January 2014 to June 2015 (17 months)   - < 10% - 10-33% - 34-66% - 67-100% - Unknown | 4/24  7/24  6/24  6/24  1/24 | 16.6%  29.2%  25.0%  25.0%  4.2% |
| SBMT is   - mandatory for the participants - voluntary for the participants - depends on the profession of the participants | 16/24  4/24  4/24 | 66.6%  16.6%  16.6% |
| SBMT counts towards participant’s working hours   - yes - depends - no | 20/24  4/24  0/24 | 83.3%  16.6%  - |
| Participants enjoy protected time for the duration of SBMT   - yes - depends - no | 12/24  2/24  10/24 | 50.0%  8.3%  41.7% |
| **Instructors^¥^** | | |
| Is SBMT part of your job description   - yes - no | 22/71  49/71 | 31.0%  69.0% |
| What is the percentage of workload for simulation according to the contract   - < 10% - 10-20% - >20% | 64/71  5/71  2/71 | 90.1%  7.0%  2.8% |
| How much time is needed from instructors in excess   - none - some excess hours - 1 to 5 hours per month - 6 to 10 hours per month - 11 to 20 hours per month - more than 20 hours per month - unknown | 16/71  50/71  39/71  4/71  5/71  2/71  5/71 | 22.5%  70.4%  54.9%  5.6%  7.0%  2.8%  7.0% |
| Do instructors enjoy protected time for the training   - yes - no | 31/71  40/71 | 43.7%  56.3% |

* Some institutions/units use both;

€ Inter-professional: at least 2 professions including physicians and nurses; multidisciplinary: at least 2 different disciplines including pediatricians, anesthetists, pediatric intensivists, pediatric surgeons, pediatric cardiologists;

£ included are all other health care providers except physicians;

¥ total of 71 instructors are engaged in SBMT at 24 units
